# Neurophysiological predictors of deep learning based unilateral upper limb motor imagery classification

**DOI:** 10.3389/fnhum.2025.1617748

**Published:** 2025-07-04

**Authors:** Justin Sonntag, Lin Yu, Xilu Wang, Thomas Schack

**Affiliations:** ^1^Neurocognition and Action - Biomechanics Research Group, Faculty of Psychology and Sports Science, Bielefeld University, Bielefeld, Germany; ^2^Computer Science Research Centre, University of Surrey, Guildford, United Kingdom

**Keywords:** motor imagery, resting state, EEG, brain-computer interface, deep learning, machine learning

## Abstract

**Introduction:**

Motor imagery-based brain-computer interfaces (BCIs) are a technique for decoding and classifying the intention of motor execution, solely based on imagined (rather than executed) movements. Although deep learning techniques have increased the potential of BCIs, the complexity of decoding unilateral upper limb motor imagery remains challenging. To understand whether neurophysiological features, which are directly related to neural mechanisms of motor imagery, might influence classification accuracy, most studies have largely leveraged traditional machine learning frameworks, leaving deep learning-based techniques underexplored.

**Methods:**

In this work, three different deep learning models from the literature (EEGNet, FBCNet, NFEEG) and two common spatial pattern-based machine learning classifiers (SVM, LDA) were used to classify imagined right elbow flexion and extension from participants using electroencephalography data. From two recorded resting states (eyes-open, eyes-closed), absolute and relative alpha and beta power of the frontal, fronto-central and central electrodes were used to predict the accuracy of the different classifiers.

**Results:**

The prediction of classifier accuracies by neurophysiological features revealed negative correlations between the relative alpha band and classifier accuracies and positive correlations between the absolute and relative beta band and classifiers accuracies. Most ipsilateral EEG channels yielded significant correlations with classifier accuracies, especially for the machine learning classifier.

**Discussion:**

This pattern contrasts with previous findings from bilateral MI paradigms, where contralateral alpha and beta activity were more influential. These inverted correlations suggest task-specific neurophysiological mechanisms in unilateral MI, emphasizing the role of ipsilateral inhibition and attentional processes.

## 1 Introduction

Brain-computer interfaces (BCIs) are a rapidly developing field of research that offers the potential to revolutionize how we interact with technology and the world around us. BCIs are systems which have the potential to restore autonomy for individuals with motor impairments, such as those affected by paralysis or limb loss. By enabling users to control external devices through imagined movements, motor imagery-based BCIs offer a non-invasive, intuitive alternative for interactions with the environment, ranging from robotic limbs to wheelchairs, enhancing the quality of life and increasing independence (Tariq et al., 2018; Jeong et al., 2020, 2021; Palumbo et al., 2021). While invasive and partially invasive BCIs require some kind of surgery, non-invasive BCIs are easier to set up, cheaper, and have the advantage that no surgery is required, while being mostly stationary (Milan and Carmena, 2010). The paradigm of motor imagery has already been used to facilitate intuitive real-world interactions using a robotic arm (Jeong et al., 2020). Schack et al. (2014) highlighted that an individual’s imagery ability, and the structure of motor representations in memory, may shape motor imagery-related neural activity. Combined with evidence of inter-individual variability in motor imagery EEG patterns (Klimesch, 1997; Sartipi and Cetin, 2024), this suggests that subjective neurophysiological traits significantly influence BCI efficacy. Despite substantial progress, a critical challenge persists: the variability in BCI performance across individuals. To decrease this variability, also called BCI-illiteracy (Blankertz et al., 2010), it is crucial to understand the underlying neuronal mechanisms of motor imagery and how they are represented in brain imaging devices, such as the EEG, which could lead to an acceleration of the development of motor imagery based BCIs.

Motor imagery (MI) is widely used in neurorehabilitation, sports, and brain-computer interfaces (BCIs), with classification accuracies reaching up to 93.56% using deep learning methods (Arı and Taçgın, 2024). Individual differences in neurophysiological features, particularly alpha and beta EEG frequency bands, are known to influence BCI performance (Pfurtscheller et al., 2005, 2006, 1996; Blankertz et al., 2010). These features, mainly extracted from resting-state (RS) EEG, have been predictive of performance in traditional machine learning pipelines, typically incorporating common spatial pattern (CSP) analysis. However, the relationship between RS EEG markers and performance in deep learning-based BCIs remains underexplored. While deep learning models eliminate the need for handcrafted features, studies such as Kang et al. (2021) have not isolated performance by model type, limiting interpretability. Our work extends this gap by linking RS EEG features to deep learning-based BCI performance in unilateral upper-limb MI, providing model-specific relationships with neurophysiological features that support more effective BCI systems in a complex motor imagery paradigm. By this, we aim to consider modern classification methods and their prediction as well as to contribute to a better understanding of motor imagery.

The conscious process of imagining the intended content of a movement, while in contrast to that the actual movement itself is primarily performed unconsciously is defined by Lotze and Halsband (2006) as motor imagery. Furthermore, it has been reported that conscious motor imagery and unconscious motor performance share common muscle and brain activities. In addition, the subjective level of mental resources needed to imagine a movement correlates with the amount of force needed to execute that imagined movement (Lotze and Halsband, 2006). Areas of the brain that play a crucial role during motor imagery include the primary motor cortex (M1), supplementary motor area (SMA), inferior frontal gyri (IFG), precentral gyri (PcG), middle frontal gyrus (MfG), premotor cortex (PMA), parietal cortex, inferior parietal lobule, putamen, and the cerebellum (Lotze and Halsband, 2006; Mizuguchi et al., 2012). The SMA and PMA have been highlighted by several studies as essential parts of the neural network for motor imagery (Ehrsson et al., 2003; Kasess et al., 2008; Hétu et al., 2013; Barhoun et al., 2022). For the PMA, it has been reported that executions involving the fingers, toes and tongue showed activation patterns identical to those observed during imagery of the corresponding movements (Ehrsson et al., 2003). Additionally, the ventral and dorsal premotor cortices are hypothesized to play important roles in the planning and preparation of motor imagery (Hétu et al., 2013). During motor imagery, an interplay between the SMA and M1 was identified by Kasess et al. (2008), showing that the SMA suppresses M1 activity. Evidence was found for this by a rapid suppression of M1 by the SMA during motor imagery (Barhoun et al., 2022). The supplementary motor area (SMA) plays a key role in generating motor responses and mentally simulating them during motor imagery (Mizuguchi et al., 2012). It also integrates and processes visuospatial information (Hétu et al., 2013). The parietal cortex, including the supramarginal gyrus and inferior/superior parietal lobules, is highly active during sensory integration and visuomotor transformations. The basal ganglia, especially the putamen and pallidum, contribute to both motor imagery and execution (Hétu et al., 2013; Bear et al., 2018). Additionally, upper limb imagery mainly activates premotor regions, while lower limb imagery engages the SMA, cerebellum, putamen, and parietal areas (Hétu et al., 2013).

Neurophysiological parameters or features have been utilized to investigate and predict the accuracy of EEG-based BCIs (Blankertz et al., 2010; Ahn et al., 2013; Zhang et al., 2015; Kwon et al., 2020; Kang et al., 2021; Wang et al., 2022; Zhou et al., 2022). Neurophysiological predictors of BCI performance are primarily derived from the power of theta, alpha, beta, and gamma frequency bands during either an eyes-open or eyes-closed RS. Theta power showed mixed findings, while some studies found significant correlations (Ahn et al., 2013; Kwon et al., 2020), others did not (Kang et al., 2021). Generally, the eyes-open RS EEG was more predictive than eyes-closed, with higher relative alpha power generally linked to better BCI performance. Blankertz et al. (2010) demonstrated that the average PSD at 10 Hz from the C3 and C4 electrodes during a two-minute, eyes-open RS EEG correlated with motor imagery BCI accuracy (*r* = 0.53). Expanding on this, Ahn et al. (2013) analyzed a broader range of frequency bands between 4 and 70 Hz. The PSD of each frequency band was calculated and normalized by the sum of the PSD for the full range of 4–70 Hz. It was reported that relative theta power correlated negatively with BCI performance, while relative alpha power showed a positive correlation (|*r*| = 0.5). These correlations were strongest at C3 and C4, with theta power being particularly significant in frontal and posterior-parietal regions, while the alpha band showed significant correlations for all brain areas (Ahn et al., 2013). Further investigating pre-recorded RS EEG, Zhang et al. (2015) examined the PSE of the 0.5–14 Hz range and found the highest correlation at C3 for eyes-closed RS (*r* = 0.65, *p* < 0.01). Similarly, the alpha band exhibited the highest mean power amplitudes at C3 and C4, reinforcing previous findings. The dominance of the C3 electrode was attributed to the participants right-handedness and the corresponding left hemisphere dominance for motor skills. Additionally, the alpha band showed the highest mean power amplitudes at both the C3 and C4 electrodes (Zhang et al., 2015). Kwon et al. (2020) assessed the relationship between eyes-open and eyes-closed RSs in motor imagery tasks, finding that individuals with higher BCI classification accuracies (>70%) exhibited significantly higher relative alpha power in the eyes-open condition, while beta power was lower. However, unlike Ahn et al. (2013), no significant differences were found for theta power. A combination of relative alpha, beta, and theta powers of the eyes-open condition achieved the highest correlation with BCI performance (*r* = 0.71, *p* < 0.001). It was concluded that the alpha and beta frequency bands are more predictive of motor imagery classification accuracy, while theta power is not as important as alpha or beta power (Kwon et al., 2020). Wang et al. (2022) examined spectral features and complexity measures, including RPL, PSE, and Lempel-Ziv complexity (LZC). The strongest correlations with BCI performance were observed in the alpha band at the C4 electrode (*r* = 0.5 for RPL, *r* = −0.53 for PSE, *r* = −0.46 for LZC). Zhou et al. (2022) further highlighted the importance of alpha power by employing neurofeedback training, which led to an increase in relative alpha power and improved BCI performance by 8.25 ± 12.66% (*p* < 0.05). Kang et al. (2021) explored both spectral and non-linear features in RS EEG, comparing multiple CSP based machine learning classifiers and three different deep learning classifiers (ShallowConvNet, DeepConvNet, EEGNet). Since there were no significant differences between the classifiers, the accuracies of all the motor imagery classifiers were averaged. Contrary to previous studies, they reported no significant correlations for eyes-closed RS features, with only eyes-open features showing a connection to BCI performance. They found significant correlations between theta-to-beta power ratios in frontal regions (F3, *r* = 0.37, *p* < 0.001).

Building upon these findings, this study identifies a significant gap in the literature concerning the predictive neurophysiological markers of classifier performance in unilateral upper limb motor imagery paradigms. While bilateral motor imagery has been extensively studied, the unilateral context remains underexplored, particularly in relation to the distinct spectral characteristics that may influence decoding accuracy. Moreover, prior work has predominantly focused on conventional machine learning approaches, leaving the potential of deep learning-based classification underutilized. To address this, the present study integrates advanced deep learning architectures, recognized for their increasing utility in EEG-based BCI systems, to systematically investigate which resting-state EEG features (e.g., relative alpha and beta power, spectral complexity metrics) most reliably predict classifier outcomes. This approach aims to enhance the precision of motor imagery decoding by identifying robust neurophysiological predictors tailored to unilateral motor imagery tasks.

## 2 Materials and methods

### 2.1 Participants

Twenty-six participants (mean age = 24, 32; SD = 4, 66; age range = 18–37 years; 15 females) were recruited in the experiment. All of them were right-handed, as evaluated by the German version of the Edinburgh Handedness Inventory (Oldfield, 1971; *M* = 4.81, SD = 0.28). Educational backgrounds varied, ranging from Abitur (German university entrance qualification) to doctoral (PhD) level. All participants reported normal or corrected to normal vision as well as no mental illnesses or cognitive impairments. The participants had no prior experience with EEG recording, motor imagery and BCIs before except for two participants who had minimal experience with motor imagery. They were compensated by course credits if wanted. The experiment was reviewed in advance and approved by the Ethics Committee of Bielefeld University. The test subjects were also informed about the study procedure before the experiment was carried out. All participants gave their written consent under the Declaration of Helsinki before the experiment and the experimental protocol was approved by the ethics committee of Bielefeld University.

### 2.2 Study design and experiment setup

**Figure 1:**
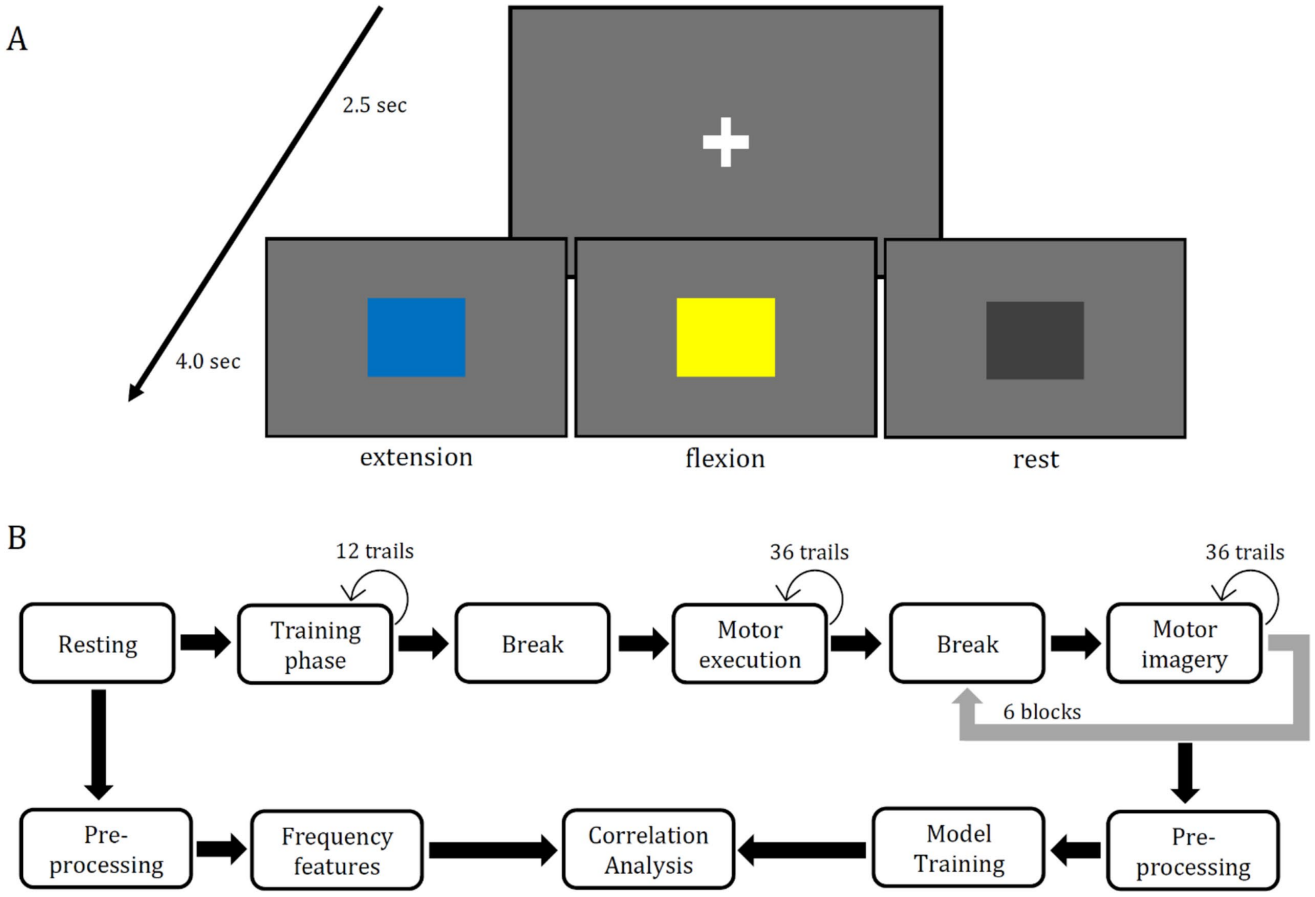
Panel **(A)** is the sequence of each trial shown, which began with a fixation cross, which was shown for 2.5 seconds. One of three colored squares followed for 4 second. The blue square indicated the extension of the right arm, the yellow square indicated the flexion of the right arm, and the gray square indicated relaxation. After these 4 seconds, the next trial started beginning with the fixation cross. The same stimuli are shown in every block of the experiment. The upper sequence in Panel **(B)** shows the sequence of data recording. The training phase consisted of 12 trials of motor execution training, the motor execution phase consisted of 36 trials and the motor imagery phase consisted of six blocks each containing 36 trials of imagery. Between each phase a 2 minute long break was planned but it could be skipped at any length by the participant to go on. The lower sequence shows the steps of data processing for the main correlation analysis. Frequency features from two RSs (eyes-open & eyes-closed) were used as well as the accuracy scores three different deep learning models (EEGNet, FBCNet, NFEEG) and two machine learning models (SVM, LDA).

The main task was to execute or to imagine the flexion or extension of the right elbow. One trial consisted of a fixation cross which was presented for 2.5 s followed by a square for 4 s. Participants were instructed to start execution/imagery the moment they perceived the square’s color and finish their execution/imagery within 4 s such that the starting position would always be the same for all trials. For every ten trials of motor imagery (5 times blue, 5 times yellow) two trials of non-task related state (2 times gray) were added. The order was randomly selected for each participant. The motor imagery experiment was separated into three phases, training, motor execution and motor imagery. The aim of the training phase was to check if the participant understood the instructions correctly. In the second phase data for the motor execution was collected. In the third phase the data for the motor imagery task was collected. The first phase consisted of 12 trials, the second phase of 36 trials and the third of 216 trials. All training trials were in one block, all motor execution trials were completed as another block, and the motor imagery trials were separated into six blocks, each block consisted of 36 trials of motor imagery. All blocks were separated by 2-min-long breaks, which could be skipped when the participant felt ready to go on. The sequence of blocks and number of blocks can be seen in Figure 1B, in the upper sequence. The current work solely used data from the motor imagery blocks for model training. The lower sequence of Figure 1B shows the processing of the data from the RSs as well as the processing of the motor imagery data for the main data analysis.

Two different unilateral upper limb movements and one non task related state condition were used in the present study within the motor imagery experiment: extension of the right elbow, flexion of the right elbow and resting (non task related state). These three conditions were indicated by different colored squares. Blue indicated extension, yellow indicated the flexion and gray indicated the non task related state. The used trial setup is illustrated in Figure 1A. The size of the presented squares was 7.5 × 7.5 cm. For the stimulus presentation a computer screen of 22 inches and the software Psychopy (v2022.2.5) were used. The screen had a refreshing rate of 60 Hz and a resolution of 1,680 × 1,050 pixels and was placed around 70 cm away from the participant. A chair was placed around 40 cm in front of the table with the screen on it. The hole setup was placed inside of a shielded cabin to prevent the distortion of EEG data and to minimize external interference. The participants were instructed to sit on the chair relaxed while their right arm should be in a natural hanging position. In advance to the main task, two three-minute RSs (eyes-open, eyes-closed) were recorded. During both, a fixation cross was shown, identical to the one in Figure 1A.

### 2.3 Procedure

The experiment procedure began by welcoming the participant and asking for their declaration of consent in a written form. Afterwards, the participants were informed about the experiment procedure in writing and verbally by the experimenter. Any questions that arose were answered at any time by the experimenter and any unclear procedures were explained again. Subsequently, a handedness questionnaire, the modified German version of the Edinburgh Handedness Inventory from Oldfield (1971) was filled out. After that, the EEG cap was prepared, and the participant entered the shielded cabin. The participants were instructed to sit on the chair relaxed while their right arm should be in a natural hanging position. In advance to the main task, the 3 min RSs (eyes-open, eyes-closed) were recorded. More information about the EEG recording can be found in Chapter 2.4. The experiment used a unilateral upper limb motor imagery paradigm similar to Marchesotti et al. (2016), Ma et al. (2020), Zhang et al. (2023), and Shi et al. (2024). The experiment focused on the extension and flexion of the right elbow while adding a non task relevant state condition. The setup was tested by the participants before the main EEG recording to ensure familiarity and comprehension of the instructions through the training phase mentioned in Chapter 2.2. Feedback was partially needed as participants finished motor execution not within the 4 s of stimuli presentation. No further instructions were given during the motor execution phase. For the motor imagery phase, participants were instructed to imagine the imagery of the associated movement in a similar way as they have performed the real movement in the earlier block before. No more instructions were given, to allow the participants to imagine the movement in a completely natural manner. The duration of the experiment varied from 2 to 2.5 h depending on the preparation of the EEG cap.

### 2.4 EEG data collection

The EEG signals were sampled at 512 Hz using the asalab software (v4.9.4 ANT neuro) and a 64-channel ANT amplifier (refa-8). The 64 electrodes were placed according to the international 10–10 system, with efforts to maintain electrode impedances below 10 kΩ. Additionally, two bipolar electrodes placed above and below the right eye and lateral to both eyes to record the electrooculography (EOG). The ground electrode served the position AFz. An online bandpass filter of DC to 1,000 Hz was employed and common reference was used during the recording.

### 2.5 Data analysis

The data analysis was carried out using the programming language Python (v3.11.6). For the analysis of the EEG data the Python module MNE (v1.7.0) was used (Larson et al., 2024). Modules for the implementation of the neural networks were TensorFlow (v2.15.0) and PyTorch (v2.1.0). For all statistical analyses the module SciPy (v1.11.3) was used.

Three different neural networks were chosen to be trained for each participant. The EEGNet from Lawhern et al. (2018) was chosen due to its flexible use in different EEG paradigms as well as it has been established as a benchmark model in the literature (Riyad et al., 2020a; 2020b; Kang et al., 2021; Song et al., 2022; Zhang et al., 2023; Shi et al., 2024). The second used neural network was the FBCNet from Mane et al. (2021). This was done to cover the special separation and analysis of the filterbanks from 4 to 40 Hz. It should be noted that the architecture of the model was modified according to Qiu et al. (2021), who applied the FBCNet with 4 spatial convolution blocks for unilateral upper limb motor imagery classification. The third neural network was the NFEEG of Arı and Taçgın (2024). This was done due to the models design to work with raw EEG data. To make a direct comparison between the literature and the current study, two machine learning classifiers were trained. Namly an SVM and an LDA were used to classify motor imagery features generated by the CSP algorithm.

Each model needed a different variation of preprocessing also shown in Table 1. For EEGNet the preprocessing followed the approach of Lawhern et al. (2018). Specifically, the data was resampled to 128 Hz and bandpass filtered between 4 and 40 Hz. In addition to these steps, eye and muscle artifacts were removed using independent component analysis (ICA), and EOG channels were excluded and the data was referenced by the common average to align preprocessing with earlier studies (Ahn et al., 2013; Aggarwal and Chugh, 2019; Wang et al., 2022). The left and right channels of the mastoids were removed afterwards. The final epoched dataset comprised a 4.5-s time series, sampled at 128 Hz, bandpass filtered between 4–40 Hz, and recorded from 62 electrodes. For FBCNet the preprocessing followed the approach of the authors from FBCNet (Mane et al., 2021). The data was resampled to 128 Hz. Nine filterbanks were extracted ranging from 4 to 40 Hz, where each filterbank consisted of a bandwidth of 4 Hz (Mane et al., 2021). Additionally, an ICA was used to remove eye and muscle artifacts, the data was referenced by the common average (Aggarwal and Chugh, 2019). EOG channels as well as the mastoid channels were removed afterwards. The final dataset consisted of nine filterbanks, each with a length of 4.5 s, sampled at 128 Hz and recorded from 62 channels. For NFEEG no preprocessing was applied except for an applied notch filter at 50 Hz. EOG and mastoid channels were removed. This was done to align with the authors intention of the model to work with no to very little earlier preprocessing (Arı and Taçgın, 2024). As both machine learning classifiers were used as a baseline, their preprocessing was reasoned to be similar as the one from EEGNet as EEGNet established itself as a baseline deep learning model in other studies (Riyad et al., 2020a; 2020b; Kang et al., 2021; Song et al., 2022; Zhang et al., 2023; Shi et al., 2024). The data was resampled to 128 Hz and filtered to contain the mu and beta frequencies, resulting in a bandwidth of 4–40 Hz. Similar to the preprocessing of the data for EEGNet and FBCNet, an ICA was applied, the common average was used as reference. EOG and mastoid channels were removed afterwards. As a final step, features were extracted from the 4.5-s long epoched data based on the CSP algorithm. Because the CSP algorithm is intended to maximize the differences between two classes, a one-vs-the-rest approach was used for each class individually (Aggarwal and Chugh, 2019).

**Table 1:** Preprocessing steps for each model.

Model	Elektrodes	Sampling rate	Epoch length	ICA	Filter	Filterbanks	Reference	CSP features
EEGNet	62	128 Hz	4.5 s	Yes	4–40 Hz Bandpass	No	Common average	No
FBCNet	62	128 Hz	4.5 s	Yes	4–40 Hz Bandpass	9 filterbanks, 4 Hz banksize, no overlap	Common average	No
NFEEG	62	512 Hz	4.5 s	No	50 Hz notch filter	No	None	No
SVM	62	128 Hz	4.5 s	Yes	4–40 Hz Bandpass	No	Common average	Yes
LDA	62	128 Hz	4.5 s	Yes	4–40 Hz Bandpass	No	Common average	Yes

Every model was trained in a three-class classification task. Each model was trained on 80% of the dataset and the other 20% were used for validation. The final reported accuracy of each model will be the average of a fivefold cross validation. Cross-entropy loss was selected as the loss function, and the Adam algorithm as the optimizer. EEGNet and FBCNet were trained on a 8-batch size and a learning rate of 1e-4 while NFEEG was trained with a batch size of 8 and a learning rate of 1e-5. All models were trained for 500 epochs.

The preprocessing of the RSs was identical. First, each RS was epoched to a length of 180 s, and then bandpass filtered from 4 to 40 Hz. In the next step, eye and muscle artifacts were removed using ICA, and EOG channels. The data was referenced by the common average (Ahn et al., 2013; Wang et al., 2022). The mastoid channels were removed afterwards. To gain the PSD of each electrode the Welch’s method (Welch, 1967) was used, which is a periodogram-based spectral estimation method, using the fast Fourier transform. The absolute power of the frequency bands, i.e., 8–13 Hz (alpha), 13–30 Hz (beta) and the RPL of each frequency band were used for further statistical analysis. The RPL for each frequency band at each electrode was calculated by dividing the power of each band by the total power across the 4–40 Hz frequency range at that electrode (Kim and Im, 2018; Wang et al., 2022).

Table 2 presents the hyperparameters used for each model. Hyperparameter values were not unified across models, as the configurations were chosen based on prior findings and recommendations from the respective authors, reflecting what was reported to yield the best performance (Lawhern et al., 2018; Ma et al., 2020; Arı and Taçgın, 2024). For the classical machine learning models, the SVM was configured with a regularization parameter C =1, an RBF kernel, a gamma value set to ‘scale’, and a tolerance of 1e-3. The LDA model employed the SVD solver with a tolerance of 1e-4. For the deep learning models, EEGNet and FBCNet were both trained using the Adam optimizer with a learning rate of 1e-4, a batch size of 32, and 500 training epochs. EEGNet used ELU as the activation function with a dropout rate of 0.5, while FBCNet applied the Swish activation function without dropout. Both models included batch normalization. NFEEG, on the other hand, used a higher learning rate of 1e-3, a smaller batch size of 8, and also trained for 500 epochs. It employed ELU activation with a dropout rate of 0.5 and batch normalization. The number of trainable parameters for EEGNet, FBCNet, and NFEEG were approximately 2,963, 2,775, and 1,260,545, respectively.

**Table 2:** Hyperparameters for deep learning models.

Model	Learning rate	Batch size	Epochs	Trainable parameters	Optimizer	Loss function	BatchNorm	Dropout	Activation function
EEGNet	1e-4	32	500	2.963	Adam	Cross Entropy Loss	Yes	0.5	ELU
FBCNet	1e-4	32	500	2.775	Adam	Cross Entropy Loss	Yes,	Non	Swish
NFEEG	1e-3	8	500	1.260.545	Adam	Cross Entropy Loss	Yes	0.5	ELU

The statistical analysis used both prior recorded RSs to get the PSD (in μV^2^) and the RPL (in %, to reduce inter-individual power amplitude variability) of each frequency band from each electrode (Ahn et al., 2013; Zhang et al., 2015; Wang et al., 2022). Based on the findings of Kwon et al. (2020), Kang et al. (2021), Zhou et al. (2022), and Wang et al. (2022), the present study focused on the alpha and beta frequency bands, as well as the frontal, fronto-central, and central electrodes. A Pearson correlation analysis between each RS value and the accuracy of each classifier was conducted to find out whether there is a linear relationship between these variables (Pearson, 1895). This analysis was performed despite one of the variables violating the assumption of normal distribution (Ahn et al., 2013). The classifier accuracies (in %) served as dependent variables.

Before the main analysis, outliers with a Z-score above three standard deviations were removed. For hypothesis testing the value for significance was chosen to be α=0.05 (Field, 2013). A comparison of data was thus just not significant above a *p*-value of ≤0.05 . Given the exploratory nature of this study, which aims to identify potential correlations for future research, we chose not to apply False Discovery Rate (FDR) correction at this initial stage. Our primary objective was to avoid overlooking potentially important findings that might be filtered out by FDR in this exploratory phase. Furthermore, the potential for complex dependencies among the correlated variables could affect the reliability of standard FDR procedures that often assume independence or positive dependence.

## 3 Results

**Figure 2:**
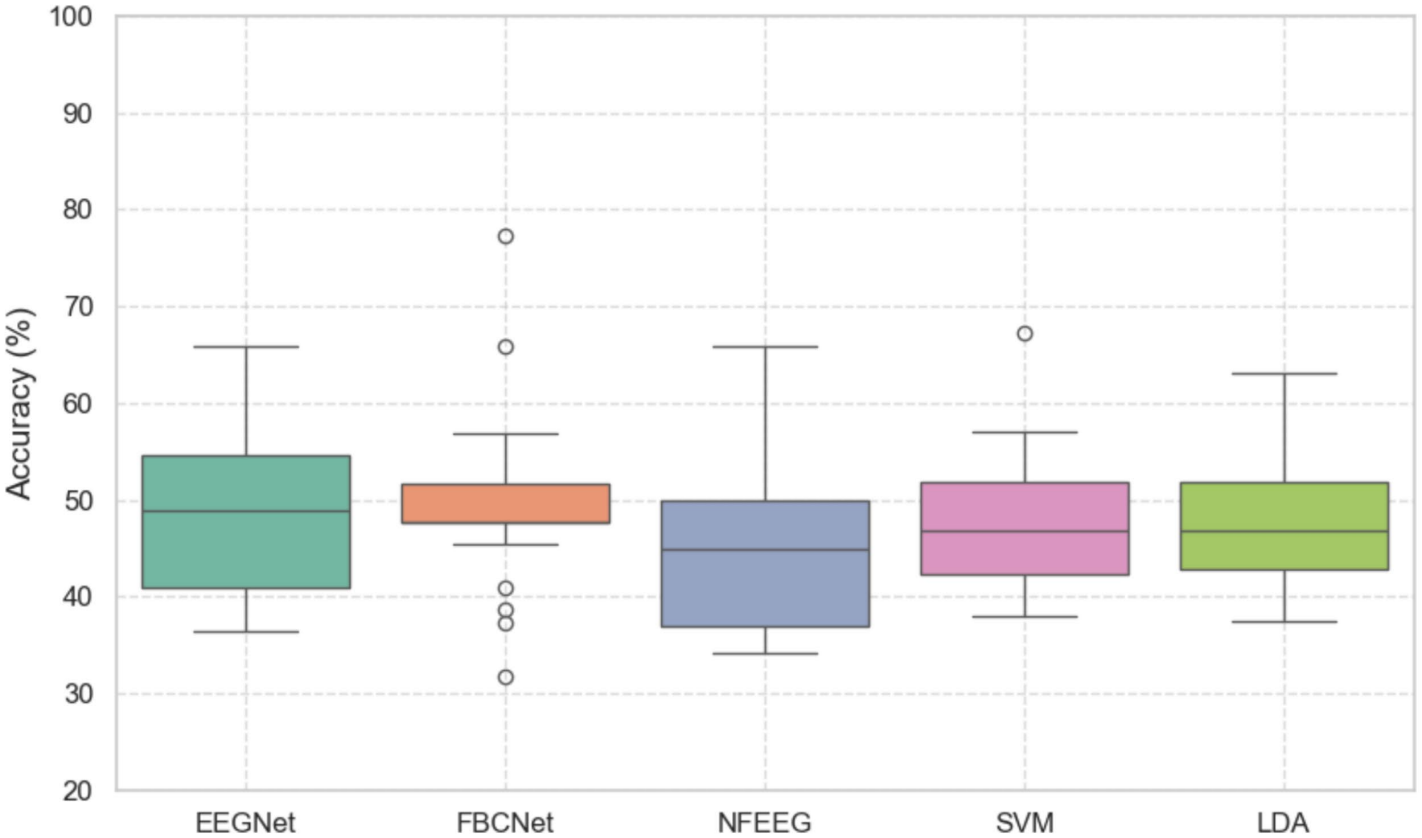
Boxplots of the accuracies (in %) of the models EEGNet, FBCNet, NFEEG, SVM and LDA. All models were trained in a three class classification problem of a unilateral upper limb motor imagery paradigm.

Five decoding methods, EEGNet, FBCNet, NFEEG, SVM and LDA, were used for an unilateral upper limb motor imagery paradigm in a three-class problem. As shown in Figure 2 boxplots of the accuracy scores for all the models are shown and did not significantly differ from each other based on a Freidman’s test (*p* > 0.05). The median accuracy scores of models were 48.86% EEGNET, 47.72% FBCNet, 44.82% NFEEG, 46.75% SVM and 46.79% LDA. Further evaluation parameters are shown in Table 3.

**Table 3:** Table of significant correlations between EEG alpha and beta frequency bands (PSD & RPL) of all electrodes and FBCNet accuracy across RSs.

Electrode	Pearson correlation coefficient	Frequency band	Power metric	Resting-state	*p*-value
FP1	0.46*	beta	PSD	eyes-open	0.018
FPz	0.49*	beta	PSD	eyes-open	0.012
Fz	0.44*	beta	PSD	eyes-open	0.023

Levels of significance are reported as follows: *p* < 0.05 (*).

To examine how and which electrodes frequency bands of RSs can be used as predictors for a unilateral upper limb motor imagery task, correlations between the PSD and RPL of each electrodes frequency bands of the RSs eyes-open and eyes-closed and the classifiers accuracies were calculated. All significant correlations of the frontal, fronto-central and central electrodes of EEGNet are shown in Table 1. In the open-eyes RS, EEGNet accuracies showed significant correlations with the PSD in the alpha band for the right frontal cortex (F8) and the right anterior frontal cortex (AF8). In the beta band, significant correlations were found at the left prefrontal cortex (FP1) and the medial prefrontal cortex (FPz). For the RPL, no significant correlations were found for the alpha band. In the beta band, significant correlations were found at the posterior parietal cortex (Pz) and the left posterior parietal cortex (P1). In the eyes-closed RS, no significant correlations were found between the PSD and EEGNet accuracies in any of the frequency bands. No significant correlations were found in the alpha or beta bands of the RPL in relation to EEGNet accuracies.

For FBCNet, no significant correlations were found for the PSD of the alpha bands in the eyes-open RS. However, in the beta band, significant correlations with FBCNet accuracies were observed at the left prefrontal cortex (FP1), medial prefrontal cortex (FPz), and medial frontal cortex (Fz). No significant correlations were found for the alpha or beta bands. In the eyes-closed RS, no significant correlations were found for the PSD of any frequency band with FBCNet accuracies. For the RPL of the alpha, and beta bands, no significant correlations were reported. Table 2 shows all significant correlations of the alpha and beta bands of the frontal, fronto-central and central electrodes of FBCNet.

For the eyes-open RS, NFEEG showed no significant correlations with the PSD of the alpha bands. However, for the beta band, significant correlations were found in the right anterior frontal cortex (AF4) with NFEEG accuracies. No significant correlations were reported for the RPL of any frequency band in the eyes-open RS. In the eyes-closed RS, no PSD of any frequency band showed significant correlations with NFEEG’s accuracies. The left frontal cortex (F1) was also significantly correlated with the RPL of the alpha band. Table 4 shows all significant correlations of the alpha and beta bands of the frontal, fronto-central and central electrodes.

**Table 4:** Table of significant correlations between EEG alpha and beta frequency bands (PSD & RPL) of all electrodes and EEGNet accuracy across RSs.

Model	Accuracy	Precision	Recall	F1-Score	Roc Auc
EEGNet	48.86	49.30	48.86	46.37	57.34
FBCNet	47.72	49.40	47.73	45.93	61.61
NFEEG	44.82	42.96	44.82	43.37	58.67
SVM	46.75	39.63	45.45	41.37	57.68
LDA	46.79	41.10	43.18	40.52	57.06

All scores are given as the median.

In the open-eyes RS, SVM accuracies were not significantly correlated with the PSD of the alpha bands. For the beta band, significant correlations were observed in the frontal areas, including the left prefrontal cortex (FP1), medial frontal cortex (FPz, Fz), right frontal cortex (F8), left anterior frontal cortex (AF7), and left fronto-central cortex (FC4), left central areas (C2, C4). For the RPL of the alpha band, significant correlations were noted in the frontal areas, including the prefrontal cortex (FP1, FP2, FPz), left frontal area (F4, F2), anterior frontal cortex (AF7, AF3, AF4, AF8), fronto-central areas (FC1, FC2), the medial central cortex (Cz), parietal areas, including the posterior parietal cortex (CPz, P3), and the occipital area (Oz). The RPL of the beta band was correlated with the frontal areas, including the medial frontal cortex (Fz), right frontal area (F8), frontal areas (F1, F2, F4), and anterior frontal areas (AF3, AF4, AF8). Additionally, significant correlations were found in the fronto-central areas (FCz, FC1, FC2, FC4), the central cortex (Cz, C2), left parietal areas (CP3, Pz, P1, P3, POz, PO3, PO5, PO7), and the occipital area (Oz). In the eyes-closed RS, no significant correlations were found between SVM accuracies and the PSD of the alpha bands. However, for the beta band, the PSD showed a significant correlation with the right prefrontal cortex (FP2). No significant correlations were found for the RPL of the alpha bands. For the beta band, the RPL was significantly correlated with the SVM accuracies in the right precentral cortex (FC4). Table 5 shows the significant correlations of the frontal, fronto-central or central electrodes for the alpha and beta frequency bands.

**Table 5:** Table of significant correlations between EEG alpha and beta frequency bands (PSD & RPL) of all electrodes and EEGNet accuracy across RS.

Electrode	Pearson correlation coefficient	Frequency band	Power metric	Resting-state	*p*-value
AF8	−0.44*	alpha	PSD	eyes-open	0.026
F8	−0.39*	alpha	PSD	eyes-open	0.048
FP1	0.43*	beta	PSD	eyes-open	0.029
FPz	0.39*	beta	PSD	eyes-open	0.049
P1	0.39*	beta	RPL	eyes-open	0.049
Pz	0.40*	beta	RPL	eyes-open	0.046

Levels of significance are reported as follows: *p* < 0.05 (*).

In the eyes-open RS, the PSD of the alpha bands were not significantly correlated with the LDA accuracies. For the beta band, significant correlations were observed at the frontal areas (FP1, FPz, Fz, F8), left fronto-central area (FC4), and central areas (C4, C2), as well as the left anterior frontal cortex (AF7). In addition, the alpha band showed significant correlations at the frontal areas (FP1, FPz, FP2, Fz, AF7, AF3, AF8, F2), fronto-central areas (FC1, FC2, FCz), central area (Cz), posterior parietal areas (P8, CPz), and the occipital area (Oz). For the beta band, significant correlations were found in the frontal areas (Fz, F4, F8, AF4, AF8, F2, F6), fronto-central areas (FC5, FC1, FC2, FC6, FC4, FCz), central areas (Cz, C4, C2), parietal areas (CP2, CPz, CP3, CP4, P3, Pz, P4, P8, P5, P1, P2), and occipital areas (O1, POz, PO3, PO4, PO5, PO6, PO7, PO8, Oz). In the eyes-closed RS, no significant correlations were found for the alpha bands. For the beta band, significant correlations were noted at the left prefrontal cortex (FP1) and the right central area (C4). No significant correlations were reported for the RPL of the alpha bands in the eyes-closed RS, except for the beta band, where a significant correlation was found at the right fronto-central area (FC4). Table 3 shows the significant correlation of the frontal, fronto-central or central electrodes for the alpha and beta frequency bands.

## 4 Discussion

### 4.1 Key findings

The results of this study demonstrated the importance of decreased absolute and relative alpha power and increased absolute and relative beta power in prior recorded RS for predicting accuracies of unilateral upper-limb motor imagery-based classifiers. Notably, the eyes-open RS exhibited more predictors compared to the eyes-closed RS. Specifically, for each of the deep learning classifiers (EEGNet, FBCNet, NFEEG), only one electrode was significantly correlated with either absolute alpha power (EEGNet) or absolute beta power (FBCNet) in the eyes-open RS. For EEGNet, the AF8 and the F8 electrode’s absolute alpha power during the eyes-open RS were negatively correlated with EEGNet’s accuracy, indicating that lower alpha power in the right lateral prefrontal cortex can serve as a medium strong predictor (AF8 *r* = −0.44; F8 *r* = −0.39) of EEGNet’s accuracy (Field, 2013). For FBCNet, the Fz electrode showed a significant medium strong correlation (Fz *r* = 0.44) between increased absolute beta power during the eyes-open RS and improved classifier accuracy. This suggests that stronger beta power in the medial and left frontal cortex during the RS can serve as a parameter to predict FBCNet’s accuracy. The accuracy of NFEEG was negatively correlated with the relative alpha power of the left frontal area (F1 *r* = −0.39) during the eyes-closed RS, indicating that lower alpha power relative to the other three frequency bands during the eyes-closed RS can serve as a predictor for NFEEG’s accuracy, also with a moderate effect size. For the machine learning classifiers, multiple channels were found to serve as predictors of their accuracies. For the SVM-based classifier, the medial frontal cortex (Fz *r* = 0.53) and the right central cortex (C4 *r* = 0.51) exhibited the strongest linear relationships with absolute beta power during the eyes-open RS and the classifier’s accuracy. For the SVM- and the LDA-based classifier, similar relevance of the right cortex was observed for predicting the accuracies, as well as the dominance of beta power. The highest significance (*p* < 0.001) for the LDA was observed for the relative beta power of the right frontal areas (F4 *r* = 0.49; F8 *r* = 0.48) and the medial frontal areas (FCz *r* = 0.50), as well as the absolute beta power of the right central cortex (C4 *r* = 0.49). A similar proportion of significant alpha and beta power correlations was observed between the SVM-based and LDA-based classifiers. In general, only a few electrodes’ frequency bands during the eyes-closed RS (e.g., relative beta power of FC4 for SVM; relative beta power of FC4 and C4 for LDA) were significant, emphasizing the importance of the eyes-open RS.

For all frontal, fronto-central, and central electrodes, deep learning and machine learning classifiers were always negatively correlated with alpha power with classifier accuracy, while beta power was always positively correlated. Furthermore, beta frequency was more frequently correlated with classifier accuracy than alpha frequency, and beta frequency also exhibited stronger correlations in terms of effect size (see Tables 5–8).

**Table 6:** Table of significant correlations between EEG alpha and beta frequency bands (PSD & RPL) of all electrodes and NFEEG accuracy across RSs.

Electrode	Pearson correlation coefficient	Frequency band	Power metric	Resting-state	*p*-value
AF4	−0.41*	beta	PSD	eyes-open	0.040
F1	−0.39*	alpha	RPL	eyes-closed	0.049

Levels of significance are reported as follows: *p* < 0.05 (*).

**Table 7:** Table of significant correlations between EEG alpha and beta frequency bands (PSD & RPL) and SVM accuracy across testing-states.

Electrode	Pearson correlation coefficient	Frequency band	Power metric	Resting-state	*p*-value
Fp1	0.65**	beta	PSD	eyes-open	< 0.001
	−0.46*	alpha	RPL	eyes-open	0.017
Fpz	0.62**	beta	PSD	eyes-open	< 0.001
	−0.49*	alpha	RPL	eyes-open	0.012
Fz	0.53**	beta	PSD	eyes-open	0.005
	0.48*	beta	RPL	eyes-open	0.014
F8	0.46*	beta	PSD	eyes-open	0.018
	0.47*	beta	RPL	eyes-open	,015
AF7	0.53**	beta	PSD	eyes-open	0.003
	−0.47*	alpha	RPL	eyes-open	0.020
	0.43*	beta	RPL	eyes-open	0.027
FC4	0.48*	beta	PSD	eyes-open	0.013
	0.46*	beta	RPL	eyes-open	0.019
C2	0.45*	beta	PSD	eyes-open	0.021
	0.39*	beta	RPL	eyes-open	0.05
C4	0.51**	beta	PSD	eyes-open	0.007
Oz	0.41*	beta	RPL	eyes-open	0.038
FP2	−0.52**	alpha	RPL	eyes-open	0.006
AF3	−0.41*	alpha	RPL	eyes-open	0.021
AF4	−0.40*	alpha	RPL	eyes-open	0.042
F2	−0.46’	alpha	RPL	eyes-open	0.019
	0.49*	beta	RPL	eyes-open	0.012
FC1	−0.39*	alpha	RPL	eyes-open	0.05
	0.40*	beta	RPL	eyes-open	0.045
FC2	−0.47*	alpha	RPL	eyes-open	0.017
	0.45*	beta	RPL	eyes-open	0.012
Cz	−0.49*	alpha	RPL	eyes-open	0.012
	0.43*	beta	RPL	eyes-open	0.029
P3	−0.39*	alpha	RPL	eyes-open	0.046
	0.43*	beta	RPL	eyes-open	0.031
CPz	−0.39*	alpha	RPL	eyes-open	0.045
Pz	0.42*	beta	RPL	eyes-open	0.031
O1	0.39*	beta	RPL	eyes-open	0.050
FCz	−0.49*	alpha	RPL	eyes-open	0.012
	0.48*	beta	RPL	eyes-open	0.013
P1	0.46*	beta	RPL	eyes-open	0.017
PO5	0.39*	beta	RPL	eyes-open	0.049
PO3	0.39*	beta	RPL	eyes-open	0.048
PO7	0.39*	beta	RPL	eyes-open	0.047
FP2	0.40*	beta	PSD	eyes-closed	0.041
FC4	0.41*	beta	RPL	eyes-closed	0.039

Levels of significance are reported as follows: *p* < 0.05 (*), *p* < 0.01 (**).

**Table 8:** Table of significant correlations between EEG alpha and beta frequency bands (PSD & RPL) and LDA accuracy across testing-states.

Electrode	Pearson correlation coefficient	Frequency band	Power metric	Resting-state	*p*-value
Fp1	0.60**	beta	PSD	eyes-open	0.001
	−0.41*	alpha	RPL	eyes-open	0.011
Fpz	0.50**	beta	PSD	eyes-open	0.009
	−0.50	alpha	RPL	eyes-open	0.009
Fp2	−0.53**	alpha	RPL	eyes-open	0.005
Fz	0.49*	beta	PSD	eyes-open	0.012
	−0.42*	alpha	RPL	eyes-open	0.032
	0.48	beta	RPL	eyes-open	0.014
F4	0.49**	beta	PSD	eyes-open	0.010
	0.43*	alpha	RPL	eyes-open	0.030
F8	0.48*	beta	PSD	eyes-open	0.014
	0.50**	beta	RPL	eyes-open	0.009
Cz	−0.46*	alpha	RPL	eyes-open	0.018
	0.47*	beta	RPL	eyes-open	0.016
C4	0.55**	beta	PSD	eyes-open	0.004
	0.44*	beta	RPL	eyes-open	0.024
FC2	−0.44*	alpha	RPL	eyes-open	0.023
	0.47*	beta	RPL	eyes-open	0.016
AF7	0.59**	beta	PSD	eyes-open	0.002
	−0.50**	alpha	RPL	eyes-open	0.010
AF8	−0.48*	alpha	RPL	eyes-open	0.001
	0.41*	beta	RPL	eyes-open	0.040
FCz	−0.48*	alpha	RPL	eyes-open	0.015
	0.50**	beta	RPL	eyes-open	0.009
Fp1	0.39*	beta	PSD	eyes-closed	0.048
C4	0.49*	beta	PSD	eyes-closed	0.012
FC4	0.39*	beta	RPL	eyes-closed	0.047

Levels of significance are reported as follows: *p* < 0.05 (*), *p* < 0.01 (**).

### 4.2 Interpretation of key findings

Our study identified a consistent pattern in which lower alpha power and higher beta power during RS EEG were associated with improved classification accuracy in a unilateral upper limb motor imagery (MI) task. This relationship held across different classifier types (EEGNet, NFEEG, SVM, LDA), suggesting that these spectral features may serve as reliable predictive markers of BCI performance. In particular, they may help identify individuals likely to experience difficulty in BCI control, commonly referred to as “BCI-illiteracy.” Previous studies have shown that alpha power typically decreases during cognitively demanding tasks and during motor imagery compared to rest (Pfurtscheller et al., 1996; Klimesch, 1997; Pfurtscheller et al., 2006). These alpha desynchronizations are more pronounced in individuals with higher cognitive capacity. Additionally, decreases in alpha power have been observed in dorsal brain regions, such as the sensorimotor cortex, and linked to enhanced selective spatial attention (Sapir et al., 2005; Siegel et al., 2008). Notably, these effects are usually localized in the contralateral hemisphere. In contrast, our findings revealed that ipsilateral sensorimotor electrodes showed stronger correlations with classification accuracy than contralateral ones. This suggests that, during unilateral upper limb motor imagery, increases in ipsilateral alpha power relative to RS may provide more distinctive and useful features for classifier learning than contralateral decreases. Supporting this, Brinkman et al. (2014) reported increased alpha-band power in the ipsilateral sensorimotor cortex during unilateral upper limb motor imagery, particularly under high movement selection demands. They proposed that alpha oscillations may reflect functional inhibition of task-irrelevant areas, helping to allocate computational resources efficiently. Similarly, Stolk et al. (2019) associated ipsilateral alpha increases in the somatosensory cortex with spatially unspecific cortical inhibition, potentially involved in suppressing irrelevant sensory inputs during motor planning and imagery.

The role of alpha power appears multifaceted. For instance, increased frontal alpha power has been linked to mind-wandering, while decreases are observed during attention-demanding tasks, including motor imagery (Ahn et al., 2013; Compton et al., 2019). Frontal alpha activity is also closely related to motor execution and observation (Babiloni et al., 2015; Wang et al., 2022), which share overlapping neural substrates with motor imagery (Lotze and Halsband, 2006). Thus, alpha oscillations during motor imagery may reflect both attentional focus and motor-related processing. In line with this, alpha (mu) rhythm suppression in motor-related cortex is a well-established marker of motor imagery (Pfurtscheller et al., 1996; 2006; Ahn et al., 2013; Zhang et al., 2015). The observed negative correlation between alpha power during eyes-open RS and motor imagery classification performance in our study may reflect a stronger relative increase in alpha power during motor imagery in participants with lower RS alpha, thereby offering stronger features for classification. Reasoned by this, our findings of alpha power increase in frontal, fronto-central, and central regions align with known patterns of inhibition in the ipsilateral primary motor cortex, supplementary motor area, and premotor cortex during unilateral motor imagery (Brinkman et al., 2014; Stolk et al., 2019). Consequently, lower relative alpha power in these regions during RS was a significant predictor of classification accuracy, emphasizing the disengagement for prefrontal and motor-related areas in effective motor imagery-based BCI control.

Frontal beta activity has been associated with top-down motor inhibition (Picazio et al., 2014) and attentional processes during RSs (Rogala et al., 2020). Beta power suppression in the frontal areas during motor imagery suggests cooperation between posterior and frontal regions for action planning (De Lange et al., 2008). The right inferior frontal cortex and motor areas exhibit beta synchronization to inhibit non-relevant information (Sacchet et al., 2015), a pattern also observed during movement cancellation, where an initial beta decrease is followed by a stronger increase (Vázquez Marrufo et al., 2001; Sacchet et al., 2015). Increased absolute and relative beta power in frontal regions has also been linked with inhibitory processes in motor control (Picazio et al., 2014). Somatosensory beta oscillations during visual cue anticipation further highlight the role of sensory processing in attentional tasks (Kilavik et al., 2013), potentially explaining the relationship between frontal beta power during eyes-open RSs and classifier accuracies (Tables 5–6, 8). The present findings demonstrate that beta power during RS, particularly over ipsilateral sensorimotor regions, show significant correlations with motor imagery classification performance for upper limb movements. This suggests that ipsilateral cortical activity contributes meaningfully to motor imagery decoding, a notion supported by previous work implicating the ipsilateral M1 and the SMA in both motor execution and imagery (Chen et al., 1997; Van Wijk et al., 2012; Schmidt et al., 2019). Importantly, beta power is characteristically high at rest and during stable postures, and decreases during motor execution and imagery in contralateral motor cortex, a process known as event-related desynchronization. Following the cessation of movement, beta power typically rebounds in what is known as post-movement beta rebound or event-related synchronization (Pfurtscheller et al., 1996). In the context of ipsilateral beta decrease, high resting beta power might reflect a predisposition for stronger beta modulation during motor imagery, making it a potentially predictive marker for unimanual motor imagery decoding. Furthermore, our data revealed a positive correlation between beta power and classification accuracy, contrasting with a negative correlation for alpha power. One plausible explanation is that alpha activity in the left sensorimotor cortex was relatively uniform across imagery conditions, thereby contributing less class-specific information. In contrast, beta power, particularly from ipsilateral channels, appeared to encode more discriminative features, possibly due to its association with proximal arm and shoulder representations (Hasegawa et al., 2017).

Notably, centrally located electrodes exhibited similar patterns of alpha and beta power during the RSs (Tables 1–3). These patterns were negatively correlated with classifier accuracies for alpha power and positively correlated for beta power. Oscillatory activity in the medial wall of the motor cortex has been associated with leg movements (Meier et al., 2008). Given these alpha and beta power distributions, it is reasonable to hypothesize that these regions contributed distinct features to the classifiers, potentially reflecting the need to inhibit leg movements during the task. This interpretation aligns with prior work showing that successful motor imagery involves active suppression of non-relevant motor regions (Pfurtscheller et al., 1997).

### 4.3 Comparison to previous studies

Comparing the results of this study to the existing literature, some findings align, while others show discrepancies. Regarding the range of correlation coefficients reported in the literature, the findings of the present study are consistent. The strongest correlation coefficient reported in the literature was by Zhang et al. (2015) for the absolute alpha power of the C3 electrode (*r* = 0.65), while the weakest was reported by Kang et al. (2021) for the beta power of the F3 electrode (*r* = 0.37). In the current study, focusing on alpha and beta power in the frontal, fronto-central, and central areas, the strongest correlation coefficient was observed for the absolute beta power of the Fz (*r* = 0.53) electrode during the eyes-open RS, which predicted SVM accuracy. Notable findings also include the absolute beta power of the C4 electrode for both SVM (*r* = 0.51) and LDA (*r* = 0.55). Another aspect where this study aligns with the literature is that electrodes around the sensorimotor areas (C3, C4) exhibited the strongest effect sizes for correlations with machine learning classifier accuracies. This is consistent with findings from prior studies (Blankertz et al., 2010; Ahn et al., 2013; Zhang et al., 2015; Kwon et al., 2020; Kang et al., 2021; Wang et al., 2022; Zhou et al., 2022). However, while these studies primarily utilized bilateral or combined upper- and lower-limb motor imagery, the present study identified mostly ipsilateral and medial located electrodes as predictors for the classifiers. Unlike many previous studies that employed bilateral motor imagery, our findings emphasize the predictive role of ipsilateral electrodes, likely due to the unilateral nature of the task (Chapter 4.2).

Besides the importance of ipsilateral electrodes compared to contralateral electrodes, a key finding of this study is that all alpha-related features were negatively correlated, while all beta-related features were positively correlated. This observation stands in direct contrast to the existing literature, where alpha power features were consistently reported as positively correlated and beta features as negatively correlated (Blankertz et al., 2010; Ahn et al., 2013; Zhang et al., 2015; Kwon et al., 2020; Kang et al., 2021; Wang et al., 2022; Zhou et al., 2022). Thus, the present study reports inverted effects for alpha and beta-related features across both deep learning and machine learning classifiers, as well as for both RS conditions.

One important factor is the paradigm used in this study compared to those in the literature. The paradigm employed here, focusing on unilateral upper limb motor imagery, may reveal a reversed mechanism compared to the paradigms used in previous studies, where alpha and beta correlations are consistent with traditional findings. This difference suggests that unilateral motor imagery paradigms may inherently exhibit an inverted relationship between alpha and beta correlations for ipsilateral electrodes. In addition, an increase in alpha power from eyes-open to eyes-closed RS was observed in this study, a finding that is consistent with Kwon et al. (2020) and supports the validity of the present data. Additionally, Wang et al. (2022) reported stronger alpha power correlation coefficients during eyes-open RS compared to eyes-closed states, attributing this to the influence of the occipital alpha band (~10 Hz) power. This aligns with the present study, where alpha band features were exclusively significantly correlated during the eyes-open RS, except for NFEEG.

Taken together, the findings of this study suggest an inverted relationship between alpha and beta power in RS EEG and classifier performance in unilateral upper limb motor imagery, compared to the patterns typically observed in bilateral motor imagery paradigms. Specifically, alpha power features were negatively, and beta power features positively, correlated with classification accuracy—opposite to the trends consistently reported in prior studies (Blankertz et al., 2010; Ahn et al., 2013; Zhang et al., 2015; Kwon et al., 2020; Kang et al., 2021; Wang et al., 2022; Zhou et al., 2022). This inversion may reflect inhibitory processes in the ipsilateral motor cortex, with increased alpha and decreased beta activity indicating suppression of non-task-relevant motor areas. While previous work frequently identified both C3 and C4 as key contributors to classification performance in bilateral imagery tasks, the present study found significant correlations only at C4, suggesting a more ipsilateral-dominant pattern. This may indicate that unilateral motor imagery elicits more distinctive neural activity in ipsilateral regions, whereas bilateral tasks may engage more symmetric or overlapping contralateral areas, reducing classifier separability. These results highlight how the motor imagery paradigm, unilateral vs. bilateral—can fundamentally alter the relationship between RS spectral features and decoding performance. They also underscore the importance of considering task design and laterality when interpreting or comparing BCI studies.

### 4.4 Limitation

The findings of our work are limited by some reasons. First, classifier accuracies were distinctly lower in the present unilateral upper-limb motor imagery paradigm. While our study yielded accuracy values ranging from median values of 44.82 to 48.86%, classic motor imagery paradigms (involving multiple body parts), have reported accuracies ranging from less than 40 to 100% (Kwon et al., 2020; Kang et al., 2021; Wang et al., 2022). This might be reasoned by the fact that earlier research in this field focused on classical motor imagery paradigms and lower accuracies across all types of classifiers were expected due to the more complex nature of the unilateral upper-limb motor imagery paradigm. Another limitation is the training size of 216 trials per participant, which may have been insufficient for the classifiers to fully learn the complexity of the task. Nevertheless, it cannot be assumed that the classifiers did not learn reasonably well, as the accuracies achieved in this study were higher compared to other studies employing similar motor imagery paradigms, such as those by Ma et al. (2020) and Zhang et al. (2023). It can be stated that larger datasets, such as those used in Shi et al. (2024), would more likely have resulted in improved accuracies. Data augmentation methods were considered as a possible solution to artificially increase the training size. However, due to the lack of a golden standard for EEG data augmentation and the model-dependent nature of existing augmentation methods, no data augmentation was applied in this study. Moreover, EEG data augmentation is an independent and ongoing field of research (Rommel et al., 2022), which is out of scope of this study. An additional limitation is that all assumptions regarding why and how specific electrodes and frequency bands serve as valid predictors for motor imagery-based classifiers require further investigation. The non-linear mapping of classifiers from EEG data to motor imagery classes necessitates methods from explainable artificial intelligence (XAI) to better understand which features were truly important for classification. Therefore, these assumptions must be validated through investigations of what classifiers learn and how these features depend on each other. Such studies could provide greater support for why and how certain electrodes and specific frequency bands of RSs are valid and reliable predictors. A fourth limitation of this work is the inability to fully control whether participants truly engaged in motor imagery during the experiments. Motor imagery inherently relies on participants’ compliance and cognitive engagement, making it challenging to verify the accurate execution of imagined movements.

It must be noted that no false discovery rate (FDR) correction was applied, given the exploratory nature of this study. As a result, the possibility of false-positive correlations cannot be entirely excluded. However, since consistent and generalizable patterns were observed across all classifiers, it can be cautiously assumed that lower alpha power and higher beta power in ipsilateral electrodes may serve as distinctive neurophysiological predictors for the classification of unilateral upper limb motor imagery, though these findings warrant further validation. What can be stated with greater confidence is that contralateral electrodes, typically associated with higher alpha and lower beta power, did not contribute significantly to classifier predictions. Therefore, this study provides novel insights into the neurophysiological underpinnings and classification of motor imagery, specifically in the context of unilateral upper limb tasks.

### 4.5 Future directions

Future research should incorporate methods from explainable artificial intelligence (XAI) to better understand what machine learning classifiers have learned. Techniques such as SHAP values (Lundberg and Lee, 2017) or LIME (Ribeiro et al., 2016) can provide insights into feature contributions, while Grad-CAM (Selvaraju et al., 2016) and layer-wise relevance propagation (Bach et al., 2015) can be used to interpret CNN-based models. Consequently, RS based features could be combined with the results of XAI techniques to better understand BCI-illiteracy. Another critical factor in motor imagery research is the availability of high-quality and high-quantity EEG data. While prolonged recording sessions may reduce data quality due to participant fatigue and inattention, conducting multiple shorter sessions could help mitigate this issue and may also introduce learning effects across sessions. Thus, future studies should investigate how learning effects over multiple recording sessions influence motor imagery classification performance.

In scenarios where data remains limited, data augmentation techniques may offer a viable solution to enhance model generalization. Future research should explore various augmentation methods tailored specifically to unilateral upper-limb motor imagery paradigms, as no gold standard currently exists (Rommel et al., 2022). Moreover, the incorporation of attention mechanisms into classifier architectures may enhance performance. For instance, Shi et al. (2024) demonstrated that integrating SE-attention into an EEGNet-based model improved classification accuracy. Beyond performance gains, attention mechanisms could also aid in identifying meaningful patterns in EEG data, contributing to a deeper understanding of the neural correlates of motor imagery.

## 5 Conclusion

Notwithstanding these limitations, this work provides valuable insights into the prediction and usability of deep learning and machine learning approaches for BCIs based on neurophysiological parameters. This study highlights a difference between deep learning and machine learning approaches in terms of the number of feasible electrodes and frequency bands for predicting BCI illiteracy. All deep learning approaches used for unilateral upper-limb motor imagery classification demonstrated a linear relationship between the relative/absolute PSD of one frontal electrode’s frequency band and classifier accuracy. In contrast, machine learning approaches showed that multiple electrodes from the frontal, fronto-central, and central cortex could serve as predictors for classifier accuracy. In addition, the significant correlations observed from the eyes-open RS across all classifiers highlight the importance of this state compared to the eyes-closed RS, although the latter might also serve as a source of predictors.

This study investigated neurophysiological predictors of two RSs to identify linear relationships with motor imagery classifier accuracies. The results revealed a negative linear relationship between relative alpha band power and classifier accuracy. In addition, positive correlations were found for absolute and relative beta band power, particularly in frontal, fronto-central, and central areas during the eyes-open RS. Interestingly, ipsilateral electrode frequency bands were more likely to be correlated with classifier accuracies than contralateral ones, potentially due to the inhibition of conflicting movements and their motor plans and during motor imagery (Brinkman et al., 2014; Picazio et al., 2014; Stolk et al., 2019; Rogala et al., 2020). These findings oppose the typical patterns reported in previous studies, where alpha power is positively correlated with accuracy in similar regions and beta power shows negative correlations (e.g., Kwon et al., 2020; Kang et al., 2021; Wang et al., 2022; Zhou et al., 2022). This inversion may be attributed to the unilateral motor imagery paradigm used in the present study, compared to the multi-body-part imagery paradigms commonly used in the literature. In regard to the classifiers employed, CSP-based SVM or LDA classifiers showed multiple linear relationships, whereas deep learning-based approaches (EEGNet, FBCNet, NFEEG) only yielded significant results for one electrode in the frontal regions. Comparing deep learning and machine learning classifiers, deep learning classifiers yielded (non-significant) higher accuracies compared to machine learning classifiers, which had more ipsilateral correlations.

## Data Availability

The raw EEG data that support the findings of this study are openly available in the publication server at Bielefeld University (PUB) via the link https://doi.org/10.4119/unibi/3004681.
