# Supplementary material for: Neurophysiological predictors of deep learning based unilateral upper limb motor imagery classification
Source: Front Hum Neurosci. 2025 Jul 4;19:1617748. doi: 10.3389/fnhum.2025.1617748 (PMC12272612; doi:10.3389/fnhum.2025.1617748)
Supplement: Supplementary file 1 [file Data_Sheet_1.PDF]

Supplementary Material

1 Supplementary Figures and Tables

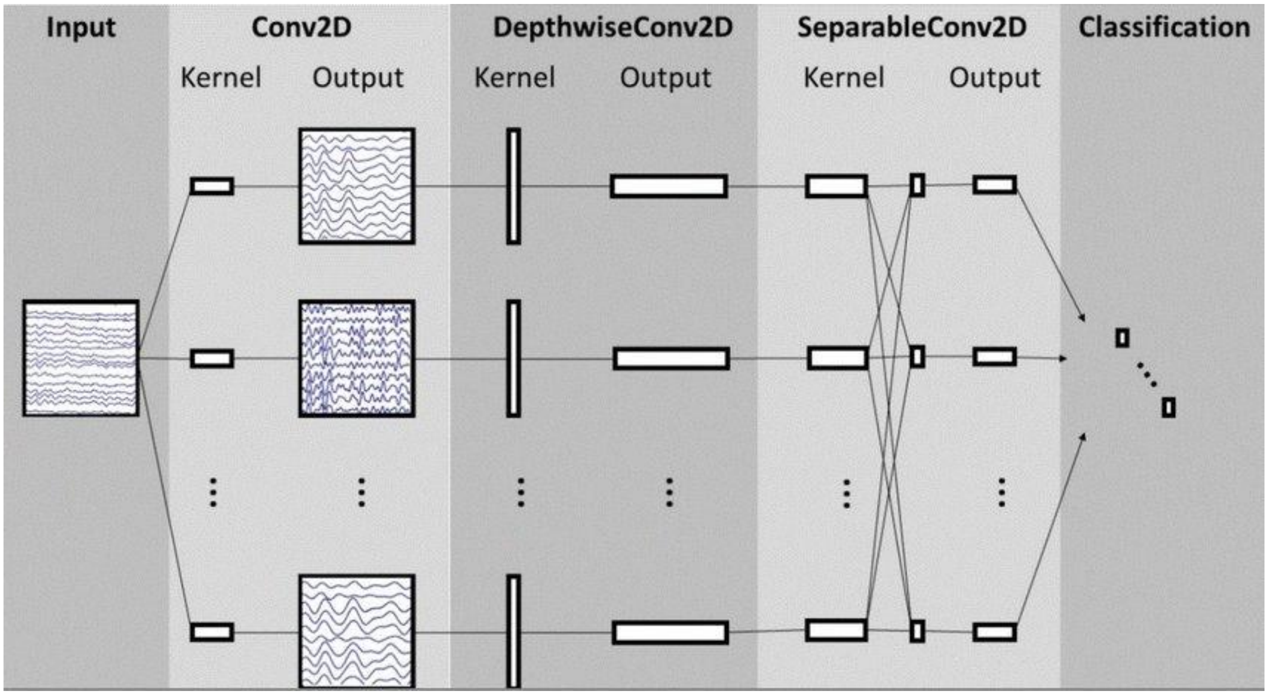

Supplementary Figure 1: The EEGNet from Lawhern et al. (2018) consists of three convolutional layers and one classification layer. The three convolutional layers apply one filter along the temporal axis by a conventional convolution, one filter along the channel axis by a depth wise convolution, and an additional filter along the temporal axis again by a separable convolution.

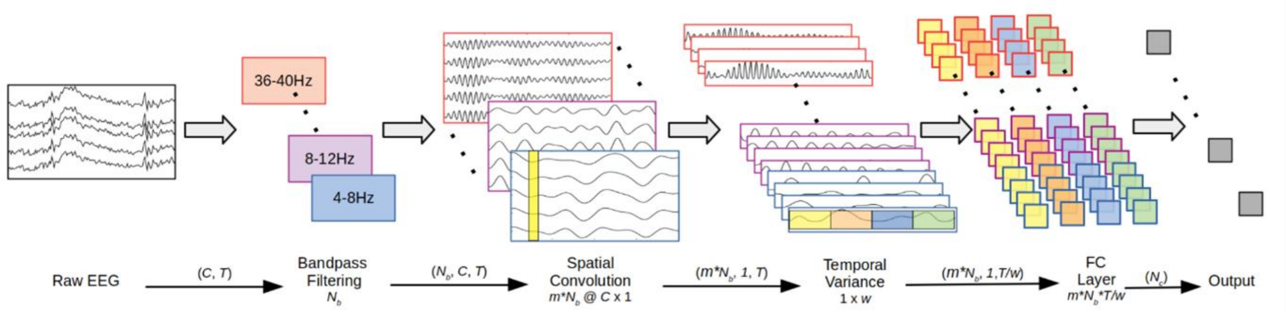

Supplementary Figure 2: Shown is the network architecture of the FBCNet from Mane et al. (2021). C represents the number of channels, T represents the number of time points, Nb represents the number of frequency bands, m represents the number of convolutional filters per frequency band and NC represents the number of output classes.

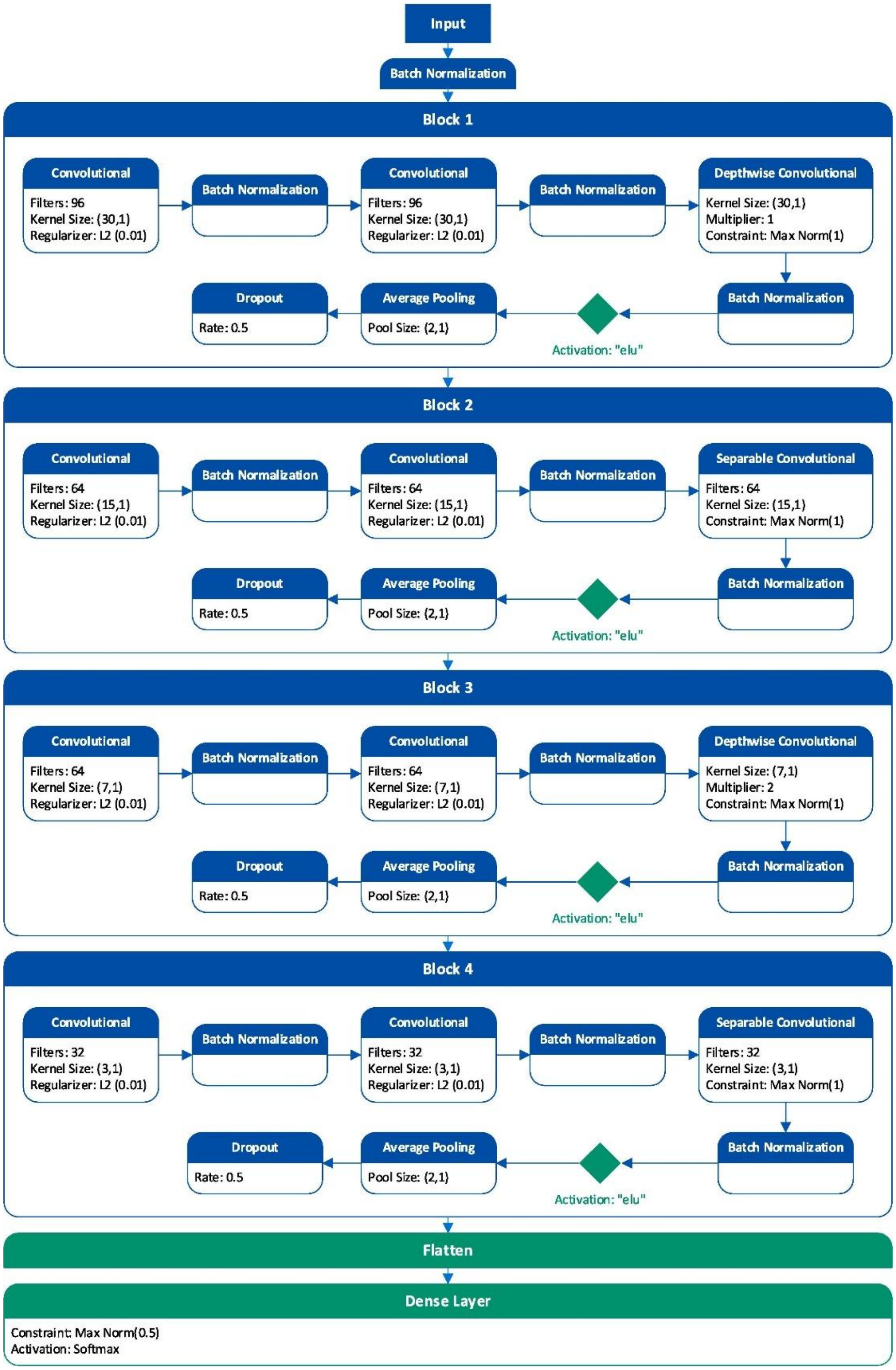

Supplementary Figure 3: The NFEEG architecture from Arı & Taçgın (2024). Their model consisted of four blocks. The block can be divided into two groups, one for temporal filtering and one for temporal and spatial filtering. The first and third block belong to group one while block two and four belong to group two.

1.1 Supplementary Tables

Supplementary Table 1. Hyperparameters for deep learning models.

Model	Learning rate	Batch size	Epochs	Trainable Parameters	Optimizer	Loss Function	BatchNorm	Dropout	Activation Function
**EEGNet**	1e-4	32	500	2.963	Adam	Cross Entropy Loss	Yes	0.5	ELU
**FBCNet**	1e-4	32	500	2.775	Adam	Cross Entropy Loss	Yes,	Non	Swish
**NFEEG**	1e-3	8	500	1.260.545	Adam	Cross Entropy Loss	Yes	0.5	ELU

Supplementary Table 2. Table of significant correlations between EEG alpha and beta frequency bands (PSD & RPL) of all electrodes and EEGNet accuracy across resting-states.

Model	Accuracy	Precision	Recall	F1-Score	Roc Auc
**EEGNet**	48.86	49.30	48.86	46.37	57.34
**FBCNet**	47.72	49.40	47.73	45.93	61.61
**NFEEG**	44.82	42.96	44.82	43.37	58.67
**SVM**	46.75	39.63	45.45	41.37	57.68
**LDA**	46.79	41.10	43.18	40.52	57.06

Note. All scores are given as the median.

Supplementary Table 2. Table of significant correlations between EEG alpha and beta frequency bands (PSD & RPL) of all electrodes and EEGNet accuracy across resting-states.

Electrode	Pearson correlation coefficient	Frequency band	Power Metric	Resting-state
**AF8**	-.44*	alpha	PSD	eyes-open
**F8**	-.39*	alpha	PSD	eyes-open
**FP1**	.43*	beta	PSD	eyes-open
**FPz**	.39*	beta	PSD	eyes-open
**P1**	.39*	beta	RPL	eyes-open

**Pz**	.40*	beta	RPL	eyes-open

Note. Levels of significance are reported as follows: p < .05 (*), p < .01 (**). (**). Ipsilateral electrodes have a light gray background.

Supplementary Table 3. Table of significant correlations between EEG alpha and beta frequency bands (PSD & RPL) of all electrodes and FBCNet accuracy across resting-states.

Electrode	Pearson correlation coefficient	Frequency band	Power Metric	Resting-state
**FP1**	.46*	beta	PSD	eyes-open
**FPz**	.49*	beta	PSD	eyes-open
**Fz**	.44*	beta	PSD	eyes-open

Note. Levels of significance are reported as follows: p < .05 (*), p < .01 (**).

Supplementary Table 4. Table of significant correlations between EEG alpha and beta frequency bands (PSD & RPL) of all electrodes and NFEEG accuracy across resting-states.

Electrode	Pearson correlation coefficient	Frequency band	Power Metric	Resting-state
**AF4**	-.41*	beta	PSD	eyes-open
**F1**	-.39*	alpha	RPL	eyes-closed

Note. Levels of significance are reported as follows: p < .05 (*), p < .01 (**). (**). Ipsilateral electrodes have a light gray background.

Supplementary Table 5. Table of significant correlations between EEG alpha and beta frequency bands (PSD & RPL) and SVM accuracy across testing-states.

Electrode	Pearson correlation coefficient	Frequency band	Power Metric	Resting-state
**Fp1**	.65**	beta	PSD	eyes-open
	-.46*	alpha	RPL	eyes-open
**Fpz**	.62**	beta	PSD	eyes-open
	-.49*	alpha	RPL	eyes-open
**Fz**	.53**	beta	PSD	eyes-open
	.48*	beta	RPL	eyes-open
**F8**	.46*	beta	PSD	eyes-open

	.47*	beta	RPL	eyes-open
**AF7**	.53**	beta	PSD	eyes-open
	-.47*	alpha	RPL	eyes-open
	.43*	beta	RPL	eyes-open
**FC4**	.48*	beta	PSD	eyes-open
	.46*	beta	RPL	eyes-open
**C2**	.45*	beta	PSD	eyes-open
	.39*	beta	RPL	eyes-open
**C4**	.51**	beta	PSD	eyes-open
**P1**	.46*	beta	RPL	eyes-open
**Oz**	.41*	beta	RPL	eyes-open
**FP2**	-.52**	alpha	RPL	eyes-open
**AF3**	-.41*	alpha	RPL	eyes-open
**AF4**	-.40*	alpha	RPL	eyes-open
**F2**	-.46'	alpha	RPL	eyes-open
	.49*	beta	RPL	eyes-open
**FC1**	-.39*	alpha	RPL	eyes-open
	.40*	beta	RPL	eyes-open
**FC2**	-.47*	alpha	RPL	eyes-open
	.45*	beta	RPL	eyes-open
**Cz**	-.49*	alpha	RPL	eyes-open
	.43*	beta	RPL	eyes-open
**P3**	-.39*	alpha	RPL	eyes-open
	.43*	beta	RPL	eyes-open
**CPz**	-.39*	alpha	RPL	eyes-open
**Pz**	.42*	beta	RPL	eyes-open
**O1**	.39*	beta	RPL	eyes-open
**FCz**	-.49*	alpha	RPL	eyes-open
	.48*	beta	RPL	eyes-open
**P1**	.46*	beta	RPL	eyes-open
**PO5**	.39*	beta	RPL	eyes-open
**PO3**	.39*	beta	RPL	eyes-open
**PO7**	.39*	beta	RPL	eyes-open
**FP2**	.40*	beta	PSD	eyes-closed
**FC4**	.41*	beta	RPL	eyes-closed

Note. Levels of significance are reported as follows: p < .05 (*), p < .01 (**). (**). Ipsilateral electrodes have a light gray background.

Supplementary Table 6. Table of significant correlations between EEG alpha and beta frequency bands (PSD & RPL) and LDA accuracy across testing-states.

Electrode	Pearson correlation coefficient	Frequency band	Power Metric	Resting-state
**Fp1**	-.60**	beta	PSD	eyes-open
	-.41*	alpha	RPL	eyes-open
**Fpz**	.50**	beta	PSD	eyes-open
	-.50	alpha	RPL	eyes-open
**Fp2**	-.53**	alpha	RPL	eyes-open
**Fz**	.49*	beta	PSD	eyes-open
	-.42*	alpha	RPL	eyes-open
	.48	beta	RPL	eyes-open
**F4**	.49**	beta	PSD	eyes-open
	.43*	alpha	RPL	eyes-open
**F8**	.48*	beta	PSD	eyes-open
	.50**	beta	RPL	eyes-open
**Cz**	-.46*	alpha	RPL	eyes-open
	.47*	beta	RPL	eyes-open
**C4**	.55**	beta	PSD	eyes-open
	.44*	beta	RPL	eyes-open
**FC2**	-.44*	alpha	RPL	eyes-open
	.47*	beta	RPL	eyes-open
	.46*	beta	RPL	eyes-open
**AF7**	.59**	beta	PSD	eyes-open
	-.50**	alpha	RPL	eyes-open
**AF8**	-.48*	alpha	RPL	eyes-open
	.41*	beta	RPL	eyes-open
**FCz**	-.48*	alpha	RPL	eyes-open
	.50**	beta	RPL	eyes-open
**Fp1**	.39*	beta	PSD	eyes-closed
**C4**	.49*	beta	PSD	eyes-closed
**FC4**	.39*	beta	RPL	eyes-closed

Note. Levels of significance are reported as follows: p < .05 (*), p < .01 (**). (**). Ipsilateral electrodes have a light gray background.
